# Molecular mechanisms of corpus callosum development: a four-step journey

**DOI:** 10.3389/fnana.2023.1276325

**Published:** 2024-01-17

**Authors:** Maria Gavrish, Angelina Kustova, Juan C. Celis Suescún, Paraskevi Bessa, Natalia Mitina, Victor Tarabykin

**Affiliations:** ^1^Laboratory of Genetics of Brain Development, Research Institute of Neurosciences, Lobachevsky State University of Nizhny Novgorod, Nizhny Novgorod, Russia; ^2^Charité Hospital, Institute of Cell Biology and Neurobiology, Berlin, Germany

**Keywords:** corpus callosum, brain development, axonal guidance and plasticity, callosal projecting neurons, neocortex, cortical midline

## Abstract

The Corpus Callosum (CC) is a bundle of axons connecting the cerebral hemispheres. It is the most recent structure to have appeared during evolution of placental mammals. Its development is controlled by a very complex interplay of many molecules. In humans it contains almost 80% of all commissural axons in the brain. The formation of the CC can be divided into four main stages, each controlled by numerous intracellular and extracellular molecular factors. First, a newborn neuron has to specify an axon, leave proliferative compartments, the Ventricular Zone (VZ) and Subventricular Zone (SVZ), migrate through the Intermediate Zone (IZ), and then settle at the Cortical Plate (CP). During the second stage, callosal axons navigate toward the midline within a compact bundle. Next stage is the midline crossing into contralateral hemisphere. The last step is targeting a defined area and synapse formation. This review provides an insight into these four phases of callosal axons development, as well as a description of the main molecular players involved.

## Introduction

One of the important aspects of the mammalian brain functioning is the exchange of information between neurons of left and right hemispheres. This process became more sophisticated during the evolution of mammals. In marsupials (infraclass Marsupialia) and monotremes (order Monotremata), interhemispheric communication is carried out through an evolutionary old structure, the anterior commissure (Flower, 1865; Smith, 1897, 1902, 1937; Abbie, 1939; Ashwell et al., 1996; Johnston, 2004; Suárez et al., 2014b). During the evolution of placental mammals (infraclass Eutheria), a new structure appeared—the corpus callosum (Latin corpus callosum, CC). In fact, it is the youngest structure in the brain of placental mammals from an evolutionary point of view (Mihrshahi, 2006; Suárez et al., 2014b). As the cerebral cortex becomes bigger in relation to the rest of the brain, and interhemispheric information flow intensifies, the CC size increases proportionally and becomes the largest commissure in humans, containing about 80% of the commissural axons of the entire brain (Finlay and Brodsky, 2006; Javed et al., 2023).

The CC is involved in various aspects of higher neuronal activity, including decision-making, social interaction, memory, and language. One of the most common congenital cerebral malformations is agenesis of the corpus callosum (ACC), which is morphologically the full or partial absence of the CC. It is often characterized by functional or behavioral disorders (Tu et al., 2009; Lau et al., 2013; Siffredi et al., 2013; Edwards et al., 2014).

**Figure 1:**
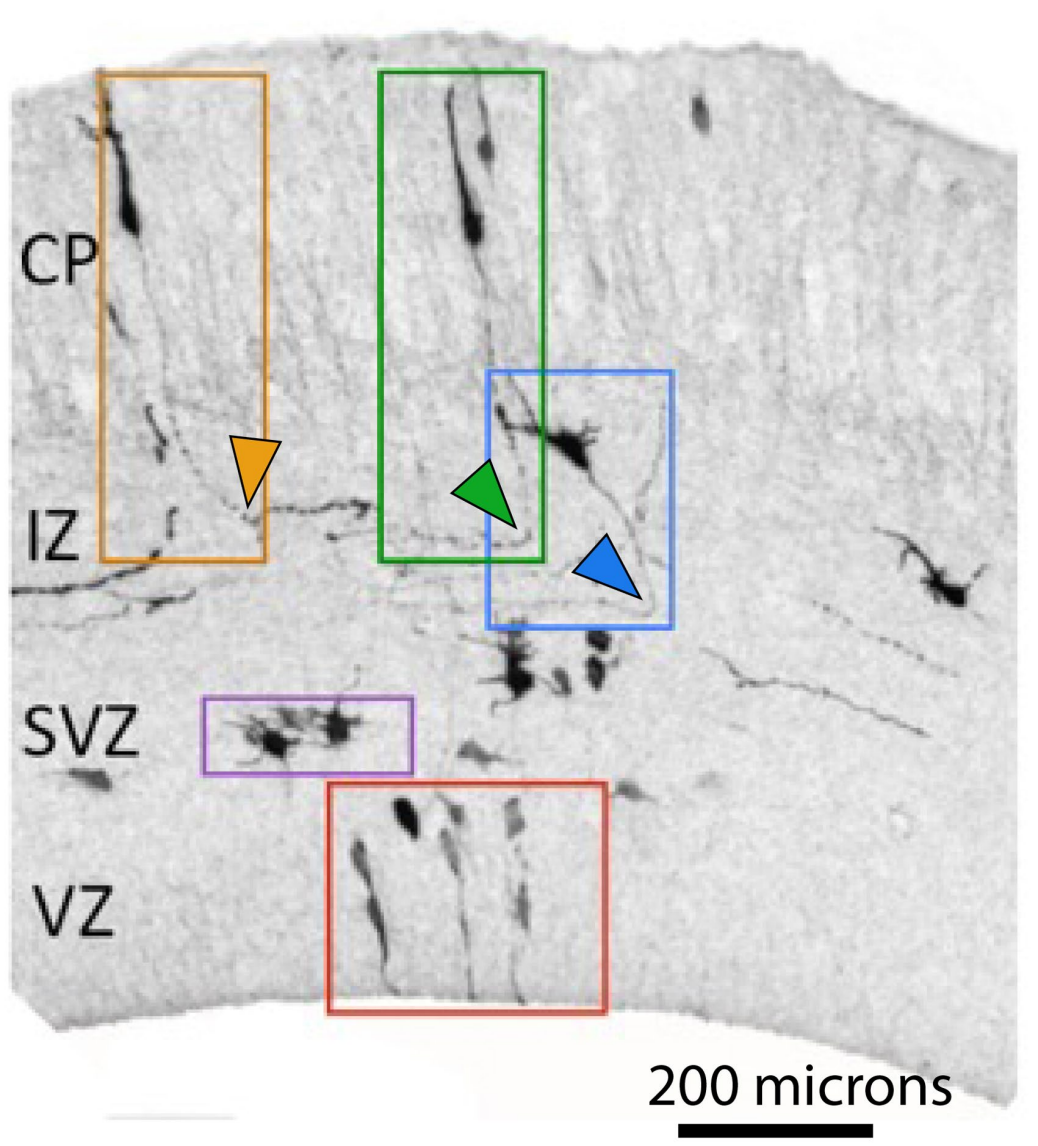
Neuronal polarization and migration during corticogenesis. Neuronal progenitors and young neurons at different stages of differentiation labeled by *in utero* electroporation with GFP in E 17.5 embryonic mouse neocortex (adopted from Bessa, 2017). Symmetric cell divisions occur in the VZ (red box depicts neuronal progenitors) while in the SVZ asymmetric cell divisions give rise to neurons (purple box depicts multipolar cells, MPC). Newborn neurons undergo polarization (blue box depicts polarized neuron with a specified axon, depicted by blue arrowhead) and subsequent migration (orange and green boxes) before finding their final position in the cortex. Orange and green arrowheads depict corticofugal and callosal axons, respectively. (CP-Cortical Plate, IZ-Intermediate Zone, SVZ-Subventricular Zone, VZ-Ventricular Zone). Note the opposite turning directions of axons depicted by green and orange arrowheads.

In this review, we describe four phases in the development of axons of callosal neurons and underlying molecular mechanisms. The first phase of CC axon navigation starts with a process known as neuronal polarization that defines an axon on one side of a neuron and a neurite known as a leading process on the opposite side. The second phase begins with the extension of the axon in a bundle with other axons toward the midline of the forebrain. The third phase, is the midline crossing. Finally, the fourth step is the targeting of an appropriate region of the neocortex and establishment of synapses with local neurons (Figure 1).

### Overview of the four phases of CC development

The neocortex is a six-layered evolutionary youngest part of the forebrain that consists of two major classes of neurons: inhibitory interneurons and excitatory projection neurons which are organized into highly specialized regions (Suárez et al., 2014b). Projection neurons are classified into several groups based on their targets (Petreanu et al., 2009; Cadwell et al., 2019). Neurons that project to targets in the contralateral hemisphere are designated Callosal Projection Neurons (CPNs) (Fame et al., 2011). These neurons are preferentially located in the superficial neocortical layers II/III and some of them are in deep layer V.

During embryonic development, all projection neurons are born in the proliferative compartment: the ventricular and subventricular zones of the dorsal portion of the telencephalon (dorsal VZ and SVZ) (Subramanian et al., 2017). At the beginning of corticogenesis the proliferative compartment consists of a single layered VZ where all neuroepethilial progenitors are in direct contact with the lumen of the lateral ventricle (Figure 1; Meyer et al., 2000; Homem et al., 2015; Montiel et al., 2016). As development proceeds, VZ progenitors transform into Radial Glia Cells (RGC) that play a dual role during development (Malatesta et al., 2003). Like neuroepethilial cells, RGCs take over the role of neuronal progenitors. Additionally, they play an important role in guiding neuronal migration. Their long fibers provide a scaffold for radially migrating neurons (Rakic, 1972; Levitt and Rakic, 1980). After onset of neurogenesis (around E13.5 in the mouse), some cells after having undergone asymmetric division migrate to the basal border of the VZ, where they form SVZ. Those of these cells that are mitotically active are designated Basal progenitors (also known as intermediate progenitors, IPs) (Noctor et al., 2008; Homem et al., 2015; Montiel et al., 2016; Wilsch-Brauninger et al., 2016).

**Figure 2:**
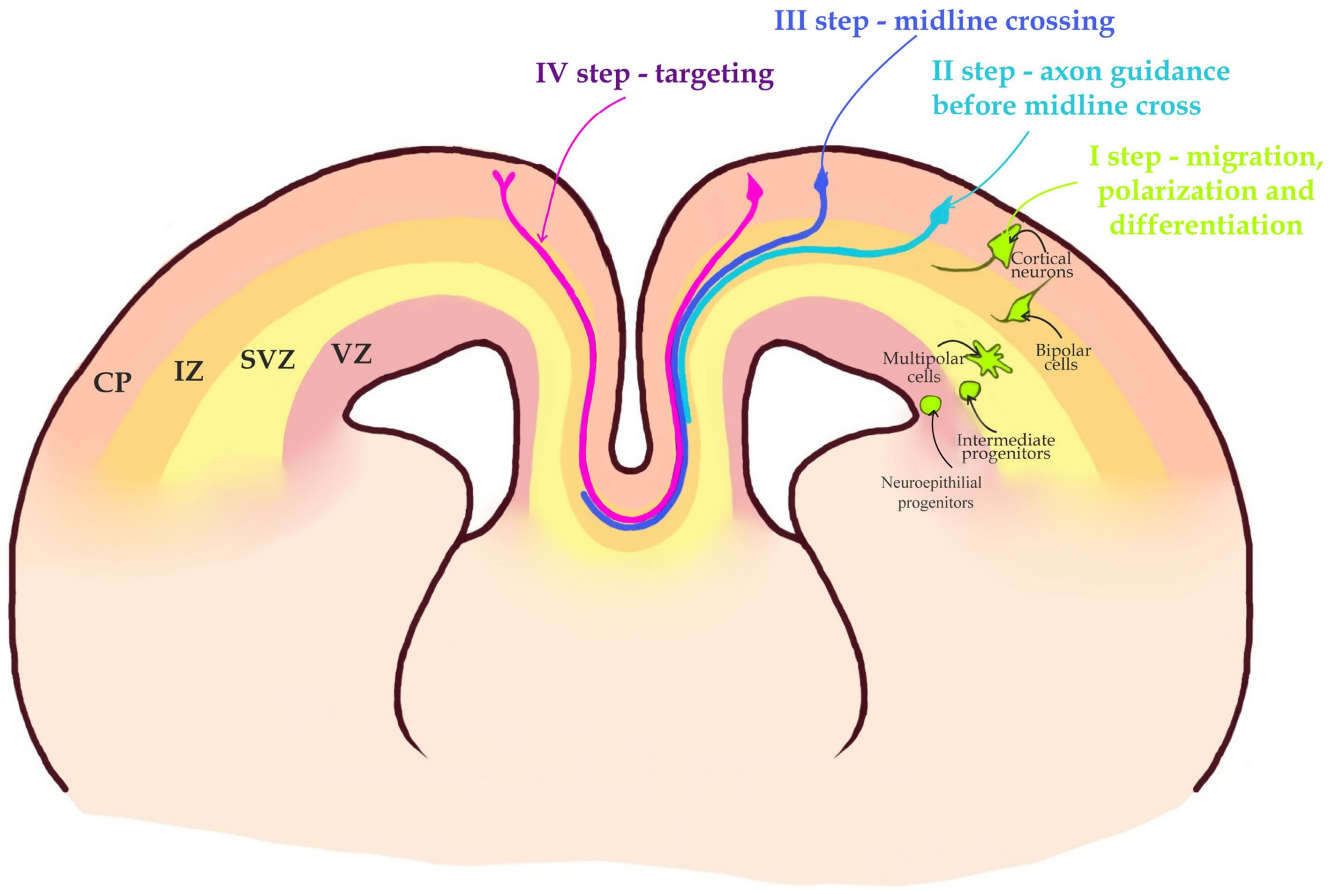
A 4-step journey of a callosal axon. I step—migration, polarization, differentiation. Intermediate progenitors generated in the Ventricular zone (VZ) by Neuroepithelial progenitors, after leaving mitotic cycle in the Subventricular Zone (SVZ) become Multipolar cells. Multipolar cells after changing their morphology to Bipolar cells become polarized. Bipolar cells migrate through Intermediate Zone (IZ), to the Cortical Plate (CP). II step—callosal axons navigate toward the midline in a bundle at the border between the SVZ and IZ. III step—the axon is attracted toward the midline and crosses it into the opposite hemisphere. IV step—axon leaves the midline and travels within the contralateral hemisphere until it reaches the target area and ascends toward the CP to establish synaptic contacts with target neurons. Note that the target area is located in the same area of contralateral hemisphere.

SVZ is an important hub for newborn neurons, this is the compartment where they start to differentiate, extend their axons and initiate radial migration along the radial glia fibers. It has been known for decades that cells after leaving mitotic cycle reside temporarily in the SVZ (“sojourn” according to Bayer and Altman, 1991). We suggest that this time is needed for neurons to interpretate the local environmental cues, unique to the SVZ. Such cues can trigger a chain of intracellular events that are eventually manifested in initiation of migration and axon extension. Cells residing in the SVZ typically have multiple small neurites and therefore are called multipolar cells (MPC) (Figure 1). One of the neurites becomes an axon, and the other one, on the opposite side of a cell becomes a leading process (Shoukimas and Hinds, 1978; Gao and Hatten, 1993; Komuro et al., 2001; Hatanaka and Murakami, 2002; Noctor et al., 2004). The remaining minor neurites are retracted, and when MPC cells reach the top of the intermediate zone (IZ, future white matter), their cell morphology changes from multipolar to bipolar (BPs) (Figure 1). This is the first phase of axon development, it is often referred as neuronal polarization. Once these two poles of a neuron are defined, it starts extending an axon while the cell soma follows the leading process that migrates along the radial glia fiber through the IZ toward the Cortical Plate (CP, future gray matter) (Figures 1, 2; Nadarajah et al., 2001; Noctor et al., 2004; Solecki et al., 2006; Sekine et al., 2011; Funahashi et al., 2014; Kon et al., 2017).

**Figure 3:**
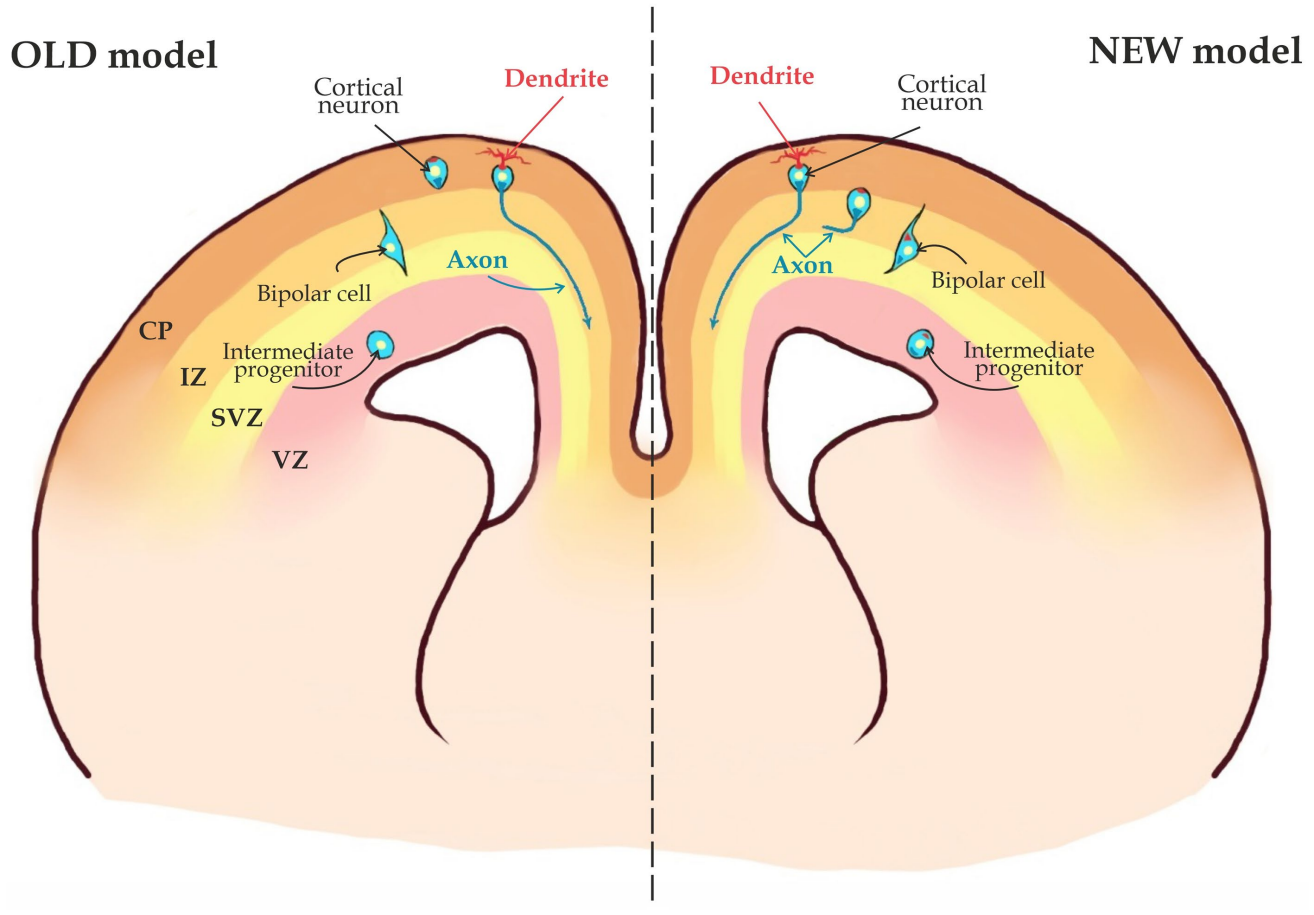
Two possible scenarios for axon specification. According to the OLD model, axons in migrating neurons are not specified and thus, axon specification occurs only when the cell has migrated into the Cortical Plate (CP). The NEW model states that axons are already specified when a young neuron is still in the Subventricular Zone (SVZ) and neuronal migration starts after the axon is specified (see the text for details).

A second step begins when a growing axon navigates within a bundle of other axons between the SVZ and IZ. The proximal tip of the growing axon, the growth cone is the part of the axon that recognizes and interprets the environmental cues. Axons within a bundle navigate together and avoid both the CP or VZ/SVZ (Figures 2, 3). Because the local cues that attract an axon in the CP of the contralateral hemisphere are identical in both hemispheres, there should exist a mechanism that prevents CP entry by a growing axon.

**Figure 4:**
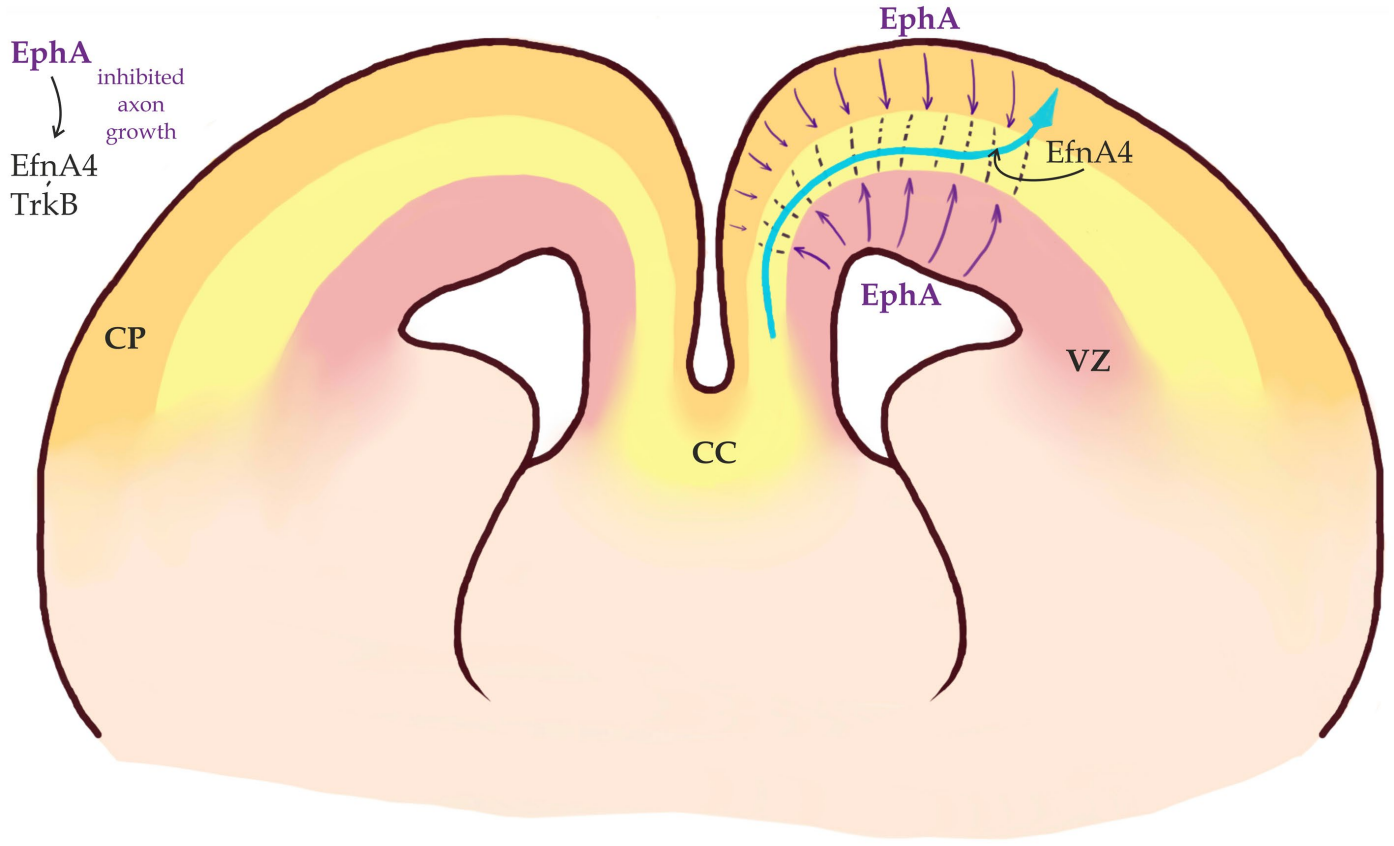
Callosal axons migrate within a permissive corridor between Ventricular Zone (VZ) and Cortical Plate (CP). EphA receptors are expressed in both VZ and CP (purple arrows) and repel EfnA4-positive callosal axons that remain bundled (modified from Yan et al., 2023).

A third step of the CPN axon navigation is the midline crossing (Figures 2, 4). This is probably the most fragile and complex among the four steps, as most mouse mutants with ACC fail to accomplish the midline crossing. This is probably because an axon attracted to the midline initially, is repelled by the midline immediately after crossing it. It means that there should be a quick switch in the molecular program within the growth cone before and after crossing (Figure 2; Fame et al., 2011; Nishikimi et al., 2013; Edwards et al., 2014; Suárez et al., 2014b).

During the final step, in the contralateral hemisphere, axons navigate as a bundle until they reach their target area, enter the CP and form synapses. This process is not random as axons of neurons residing in certain areas of the neocortex will target the same areas in the contralateral hemisphere (Figure 2; Mizuno et al., 2010; Hand et al., 2015; De León Reyes et al., 2020). Below we will review some recently identified molecular mechanisms for each phase of the CC development.

### First step—neuronal polarization and axon specification

Different types of cortical excitatory projection neurons are produced by neuronal progenitor cells residing in the VZ/SVZ of the dorsal telencephalon in a timely ordered manner, so that deeper layer (DL) neurons (layers V/VI) are born first while the upper layers (UL) neurons (layers II-IV) are generated later. While the majority of CPNs are located in UL, there are also layer V neurons that contribute their axons to corpus callosum (Kadoshima et al., 2013; Klingler et al., 2019).

Neuronal polarization—a process of transformation of a neuron from multipolar shape to bipolar is established prior to initiation of migration (Barnes and Polleux, 2009; Hatanaka et al., 2012; Sakakibara et al., 2014; Kon et al., 2017). Recently, a model has been suggested how initial polarization could cause rapid axon extention. It has been given a name «Touch&Go». According to this model, an MP cell’s small neurite starts rapidly growing and developing into an axon as soon as it contacts the pioneer axons of previously born neurons. During this process, transient axonal glycoprotein 1 (TAG1) activates the Src family kinase Lyn-induced Rac1. Rac1 activated signaling cascade contributes to axon outgrowth possibly by controlling cell skeleton dynamics (Namba et al., 2014).

**Figure 5:**
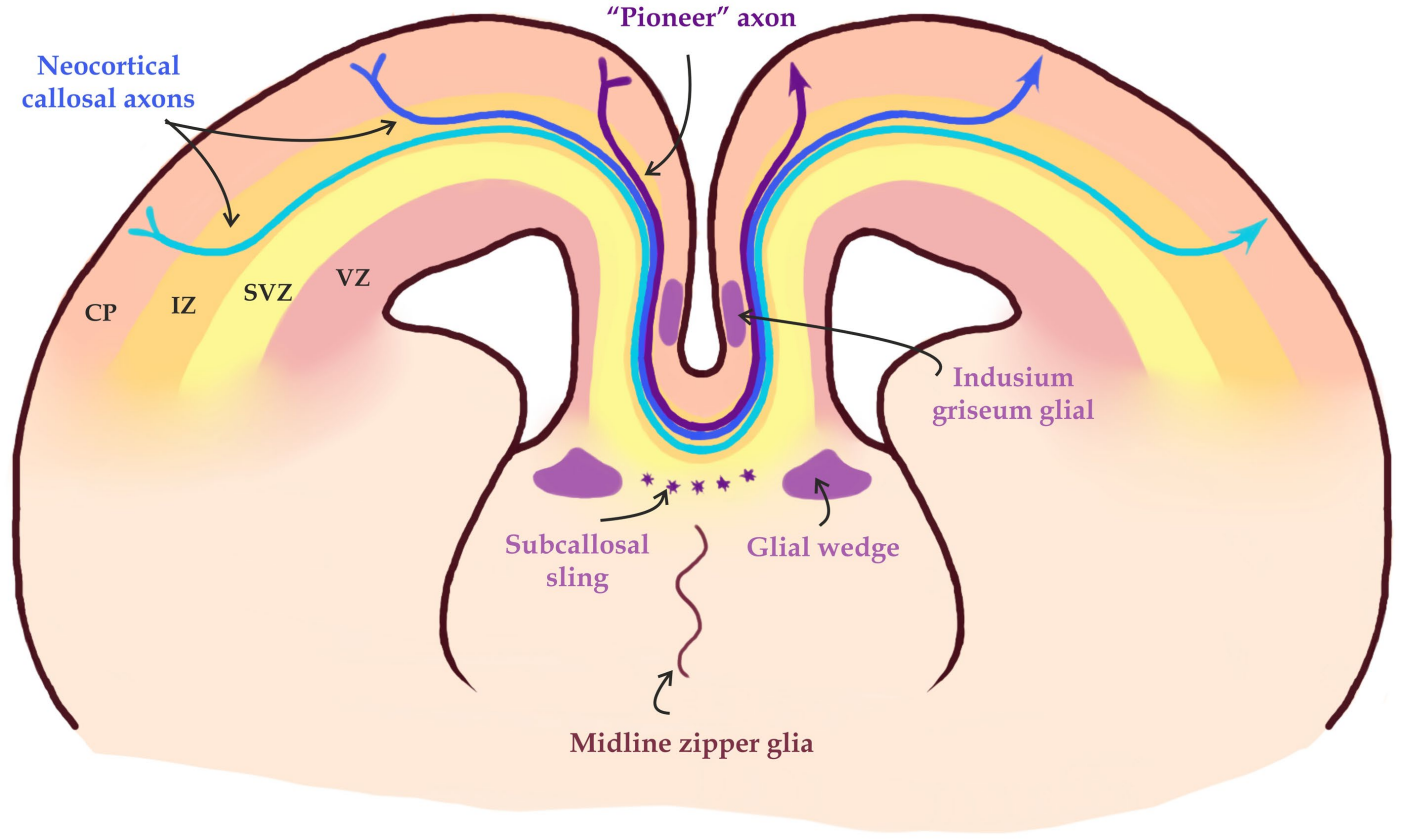
Midline crossing of callosal axons. «Pioneer» axons form a pathway for subsequent axonal projections through axon-axon interaction in part mediated by EphA3. Midline glial structures such as the glial wedge and the Indusium griseum, and neurons in the subcallosal sling help guiding axons through the midline into the contralateral hemisphere (modified from Fame et al., 2011; Song et al., 2023).

Once neurons are polarized, they start migrating into the CP (Figure 1). During this phase, an axon will be specified as whether to become a callosal axon that will navigate medially toward the midline, or to become a corticofugal axon that project to subcortical regions and will grow toward the internal capsule (Figure 1). It is not clear when exactly this decision takes place. According to an old model CPN axons are specified during or after neuron migrates toward the cortical plate (CP) through the IZ (Figure 5; Polleux et al., 1998). In this scenario a growth cone of an axon is descending toward the SVZ from IZ or CP and will either turn medially toward midline or laterally toward internal capsule. However, the dominating view now is that in the neocortex, the axon is already specified when a neuron is still in the SVZ/IZ (Figure 3; Namba et al., 2014; Bedogni and Hevner, 2021) Within the latter scenario, there might be two possibilities: According to the first one, axonal specification takes place during neuronal polarization, while a cell is still in the SVZ. In other words, once an axon is determined, it is already specified as a callosal axon. According to an alternative scenario, a nascent axon is not yet specified as a callosal or cortico-spinal axon and will not rapidly grow until the neuron is fully specified according to its cell type specific genetic program. The direction of growth in this case will be chosen once the neuron executes a respective genetic program. This might happen, when the neuron is migrating or has already reached its final position in the CP (Figure 3). In such a case a growth cone will remain immobile for an extended period of time.

#### Role of RhoA signaling

Small GTPases of Rho family-Rac1, Cdc42, and RhoA are among the main targets of the signaling pathways controlling multipolar-bipolar transition. They are the primary regulators of cell skeleton dynamics (Ohtaka-Maruyama and Okado, 2015). Numerous effectors regulate RhoA activity. Transcription factors Neurog2 and Ascl1 are activators of expression of members of Rnd subgroup of the Rho family Rnd2 and Rnd3 in migrating neurons. Their activation suppresses RhoA activity and promotes the shift from multipolar to bipolar morphology (Pacary et al., 2011). The Neurog2-Rnd2 pathway is antagonized by transcription factor Zfp238 (zinc finger protein 238), a Neurog2 downstream target that inhibits the transcription of both Neurog2 and Rnd2 (Heng et al., 2015). Rnd2 as well can inhibit axon differentiation by activating RhoA via Pragmin (Tanaka et al., 2006).

Another important player in the polarization of neurons is a transcriptional repressor RP58, which can favor the establishment of neuronal polarity by downregulating the Rnd2/Pragmin/RhoA signaling cascade. Along these lines, histone methyltransferase G9a inhibits the activity of RhoA by suppressing the expression of Lfc, a guanine nucleotide exchange factor (GEF) for RhoA (Wilson et al., 2020). The Ascl1-RNd3 pathway is also associated with extracellular signaling via the Plexin B2 receptor, which also regulates RhoA activity.

Another effector that acts upstream of RhoA is a cell adhesion molecule N-Cadherin. It plays a crucial role during the radial glia guided radial migration of neurons (Kawauchi et al., 2010; Matsunaga et al., 2017; Horn et al., 2018). When it is inactivated, both neuronal migration (Jossin and Cooper, 2011) and the transition from multipolar to bipolar morphology is disrupted (Gärtner et al., 2012). It was also shown that N-cadherin promotes the formation of the leading process by activating RhoA (Xu et al., 2015).

Another regulator of Rho in the multipolar-bipolar transition is Cdk5 cyclin-dependent kinase. It acts via p27Kip1 (cyclin-dependent kinase inhibitor 1B (CDKN1B)) and/or Mst3 (mammalian Ste20-like protein kinase 3). Cdk5 significantly reduces RhoA activity (Fame et al., 2011; Tang et al., 2014; Takano et al., 2015; Ku and Torii, 2021). Additionally, Cdk5 can phosphorylate a large amount of proteins in a cell such as Dcx (doublecortin), del1 (developmental endothelial locus-1 protein), Crmp2 (collapsin response mediator protein 2), Dbn (drebin-like protein) and p35, responsible for migration of MPs (Ohtaka-Maruyama and Okado, 2015; Shah and Rossie, 2018).

#### Transcription factors in the control of neuronal polarization

In the last two decades there have been identified several transcription factors that control axon specification in the developing neocortex. Transcriptional factor FoxG1 negatively controls the expression of Unc5D receptor in the SVZ. Unc5D is expressed in the SVZ, however, for the multipolar-bipolar transition to occur, its expression level has to be downregulated by FoxG1 in late MPs (Miyoshi and Fishell, 2012). Another transcription factor, NeuroD1 determines the formation of early MPs cells. Both NeuroD and Unc5D, seem to be targets of transcription factor Ngn2 (Cooper, 2014; Inoue et al., 2017; Mizutani, 2018). There is evidence that Ngn2 and Unc5D are also RP58 target genes (Cargnin et al., 2018). Knockout experiments of the RP58 gene have shown that an activation of both genes leads to the inhibition of the multipolar-bipolar transition (Ohtaka-Maruyama et al., 2013).

Sox9 transcription factor acts through Wwp1 and Wwp2 E3 Ubiquitin Protein Ligases as well as miR-140 microRNA to control the acquisition of axonal and dendritic polarity in neocortical neurons. Furthermore, miR-140 inhibits FYN kinase mRNA, which allows the cell to acquire BPs morphology. During neurogenesis, expression levels of Sox9 and Wwp2 are suppressed, while those of Wwp1 are increased (Ambrozkiewicz et al., 2018).

#### Transcriptional control of axon specification

Another set of transcription factors controls axon specification, − guidance of axons to their target areas. These are the transcription factors that control neuronal subtype specific genetic programs: Fezf2, Sox5, Tbr1, Satb2, and Ctip2. Inactivation of these transcription factors usually causes axon guidance disruption.

Fezf2 is expressed in NPCs as well as in the DL neurons. During postmitotic differentiation, neurons in layers V and VI begin to express, Ctip2. When Fezf2 is inhibited, Ctip2 expression is disturbed (Chen et al., 2005; Das and Storey, 2014). When Fezf is inactivated, neurons of the layers V and VI assume the phenotype characteristic for callosal projection neurons (Chen B. et al., 2008; Chen G. et al., 2008), and vice versa, the upregulated expression of Fezf2 in neurons that form callosal projections redirects their axons to subcortical targets (Molyneaux et al., 2005).

Sox5 is involved in the regulation of differentiation time of neurons in deeper layers (Lai et al., 2008). Sox5 is expressed by subcortical projection neurons in layers V and VI. Its expression is excluded from CPN neurons (Lai et al., 2008). When Sox5 is inhibited, aberrant migration patterns are observed, and neurons are not distributed into separated layers. They also begin to overexpress Fezf2 or Ctip2 factors, and occupy the deepest regions of the CP rather than their normal positions.

Satb2 is a transcription factor that controls the correct targeting of CPN neurons. If Satb2 is absent, the axons of the UL neurons are not able to form CC, and begin to extend axons to subcortical targets. Such neurons show changes in the expression of several axon guidance molecules, as well as ectopic expression of Ctip2 in CPNs, although Fezf2 expression patterns are not affected. On the other hand, overexpression of Satb2 significantly reduces the proportion of cells expressing Ctip2 and modifies the projections of DL neurons. Satb2 functions as a repressor of Ctip2 in callosal projection neurons (Arlotta et al., 2005; Alcamo et al., 2008; Britanova et al., 2008). Ctip2 repression by Satb2 seems to be direct as Satb2 binds directly to enchancer regions at the Ctip2 locus, where it modifies the chromatin configuration to a non-activated state (Gyorgy et al., 2008). It was also shown that this process is carried out with the cooperation with a co-factor Ski (Baranek et al., 2012). DOT1L is another player that can also influence the development of callosal axons via AF9 transcriptional regulator (Büttner et al., 2010). Recently, it was shown that a deficit of DOT1L can lead to a decrease in Satb2 activity causing activation of Ctip2 expression (Franz et al., 2019). INPP4B is another a regulator of the Satb2 expression. INPP4B seems to be also involved in the specification of axons that form the CC (Ji et al., 2016).

#### Extracellular signals

Another major class of molecules that can control neuronal polarization are extracellular secreted and membrane bound ligands and their receptors. They include secreted factors such as BDNF, NT-3, IGF-1, Wnt5A, TGF-β, Netrin, Reelin, and Semaphorin 3A, as well as adhesion molecules such as TAG1 and N-cadherin, and extracellular matrix components like laminin, all of which provide the signals required for neuronal polarization *in vivo*. Netrin receptor DCC can interact with Dab1 which intensifies the speed of MPs differentiation into BPs (Attardo et al., 2008; Homem et al., 2015; Dwyer et al., 2016; Zhang et al., 2018; Xing et al., 2021). The role of such factors is recently reviewed elsewhere in details (Gu et al., 2023). Above mentioned molecules are required for BP to MPC transition and therefore axon determination. However, it is not clear if any of these molecules are required for specification of callosal versus corticofugal axons. Further experiments need to be conducted in order to answer this question.

### Second step—axon navigation to the midline

#### Role of Satb2 and Ctip2 regulated cascade

Once callosal axons start navigating toward the midline, the tip of an axon-growth cone takes over the control of outgrowth direction. Growth cone is a highly dynamic structure containing a lamellipodial network that interacts with soluble, extracellular matrix or membrane bound molecules that act as either attractants or repellents (Zang et al., 2021). Callosal axon guidance is a coordinated set of interactions between attractive and repulsive stimuli that direct extending axons to the midline.

Important mechanisms of callosal navigation were discovered when investigating Satb2 and Ctip2 mutants. Published data indicate that callosal neurons of neocortical layer II-III (UL) and layer V (DL) use distinct molecular programs that control development of callosal axons downstream of Satb2. As deletion of Satb2 in the neocortex leads to an ectopic upregulation of Ctip2 in UL neurons, with a corresponding misrouting of callosally projecting neurons to subcortical targets (Britanova et al., 2008), Ctip2 controlled genetic program seems to be a default state. These two factors, that are mutually excluded in the majority of neocortical neurons, control the expression of two mutually exclusive receptors of Netrin, DCC and Unc5C. Unc5C and DCC play important, often opposite roles, in guiding axons in various systems (Kennedy et al., 1994; Keino-Masu et al., 1996; Serafini et al., 1996; Ackerman et al., 1997; Métin et al., 1997; Finger et al., 2002; Srivatsa et al., 2014). DCC can mediate an attractive response to a Netrin1 source, while Unc5C, in a DCC-dependent or-independent manner, has been shown to mediate a repulsive response (Keino-Masu et al., 1996; Serafini et al., 1996; Ackerman et al., 1997; Métin et al., 1997; Hong et al., 1999; Su et al., 2000; Finger et al., 2002; Gitai et al., 2003; Srivatsa et al., 2014). In the developing neocortex, Ctip2 and Satb2 negatively regulate Unc5C and DCC, respectively. This then affects whether neocortical axons are attracted or repelled by Netrin1. High levels of Unc5C expression, and low levels of DCC expression, instruct neurons to project toward the CC, since in the absence of either Unc5C or Netrin1, callosal axons misproject to subcortical targets. Therefore, a dynamic regulation of Unc5C and DCC by Ctip2 and Satb2 counteractions is responsible for mutually exclusive decisions in axonal navigation (Srivatsa et al., 2014). This Unc5C/DCC dependent mechanism is involved in the control of the second phase in the CC development, navigation within the ipsilateral hemisphere. Midline crossing of layer 5 axons is controlled by other molecules. Moreover, this mechanism does not seem to be decisive for CPN axons of layers II-III. Layer II-III neurons also require Satb2 for development of callosal axons. However, they do not depend on Unc5C/DCC interactions. Instead, their ability to produce callosal axons depends on Sema7A expression. Sema7A is down regulated in Satb2 deficient UL neurons and once reintroduced in Satb2 mutant brains can restore callosal axons. Interestingly, it is required cell intrinsically for very initial steps in axon development. When Sema7a is inactivated, the neurons residing in the SVZ fail to polarize and neither develop an axon, nor leading process. Therefore, these neurons fail to migrate and extend an axon (Bessa et al., 2023).

#### Role of NeuroD2/6 genes and ephrin signaling

Another pathway that controls guidance of callosal axon within the ipsilateral hemisphere depends on Neurod2 and Neurod6 transcription factors. The formation of the CC is severely impaired in Neurod2/6 double-mutant mice (Bormuth et al., 2013). Callosal axons in NeuroD2/6 mutant mice do not misdirect into other fiber tracts as observed in mice with impaired Satb2 expression. In contrast, they travel toward the midline, but these axons do not stay bundled, defasciculate and frequently enter the CP before reaching the midline. It was shown that this aberrant behavior is a consequence of Ephrin-Eph signaling disrupted in NeurD2/6 deficient mice. Ephrin-Eph interaction enables contact-mediated cell–cell interactions that often causes axon repulsion (Palmer and Klein, 2003; Xu and Henkemeyer, 2012; Cramer and Miko, 2016).

Ephrins and Eph receptors have a characteristic spatial distribution in the developing neocortex. While callosal axons express EfnA4, EphA receptors are expressed by the cells in both VZ and CP. EfnA4 expressing axons are repelled by EphA expressing cells. This repulsive interaction keeps callosal axons as a bundle and prevents them from entering both VZ and CP In the developing neocortex, these interactions are required to keep the growing axons within a “permissive corridor” at the border between the SVZ and CP (Yan et al., 2023; Figure 4). Eph signaling is also important for midline crossing (see below).

#### Slit/APP signaling

Slit/Robo signaling is essential for the appropriate guiding of both pre-and post-crossing callosal axons (Unni et al., 2012). Recently, it was shown that amyloid precursor protein (APP), a protein known to play a key role in the development of Alzheimer’s disease, is a newly identified receptor of Slit (Wang et al., 2017). In addition to other brain areas like the internal capsule, hippocampal commissure, and anterior commissure, APP is highly expressed in embryonic and neonatal callosally projecting neurons and layer V neurons. APP is responsible for transducing intracellular signaling after binding to Slit, which then mediates axon repulsion. It has been shown that double knockout of APP and its family member APLP2 in mice cause failure of callosal axons to reach and cross the midline (Wang et al., 2017).

### Third step—midline crossing

#### Midline structures and molecules involved

The CPN axons approach the midline in a steep ventral trajectory through the cingulate cortex. After traversing the cingulate cortex, CPN axons turn sharply to cross it at the corticoseptal boundary. Once axons reach the midline, they are guided by midline glial structures, such as the glial wedge, the indusium griseum and subcallosal sling (Niquille et al., 2009). Any disruptions in the development of these midline structures lead to complete or partial agenesis of the CC (Edwards et al., 2014).

These midline structures are primarily composed of glial populations, but they also contain populations of neurons which help navigate the CC axons by the secretion of guide molecules (Silver et al., 1982; Shu and Richards, 2001; Shu et al., 2003a,b; Nishikimi et al., 2013). Structures at the midline secrete two main types of axon guidance signals: long-rage and short-rage signals (Nishikimi et al., 2013; Figure 5). Short-range guidance molecules belonging to the Eph/ephrin family (Mendes et al., 2006) are important for the axon-glia interactions. EphrinB1 and ephrinB2 are expressed in the callosal fibers, whereas one of their binding partners EphB2 is present in the glial wedge. EphB1, EphB2, EphB3, and EphA4 are all expressed in callosal fibers, but their potential binding partners (ephrinB1, ephrinB2, and ephrinB3) are all expressed in midline guideposts (Liebl et al., 2003). One example of such interactions is ephrinB3 and Eph receptors such as EphB2 and EphB3. Knock-out experiments in mice have shown that these interactions are critical for midline guidance of CC fibers (Liebl et al., 2003).

The ventral invasion of CPN axons into the septum is inhibited by the release of an array of repulsive molecules such as Slit2, Draxin and Wnt5a from the glial wedge, a bilaterally symmetrical glial structure (Shu and Richards, 2001; Bagri et al., 2002; Marillat et al., 2002; Shu et al., 2003a; Keeble and Cooper, 2006; López-Bendito et al., 2007; Islam et al., 2009). The indusium griseum on the other hand is an area situated dorsal to the CC that consists of neurons and glia that expresses Slit2 and acts as a dorsal repulsive barrier for CPN axons (Shu and Richards, 2001; Figure 4).

Callosal axons express Slit receptors, Robo1 and Robo2 and Slit2-Robo1 interaction causes cortical axons to avoid both ventral and dorsal territories, forwarding them to cross the midline (De León Reyes et al., 2020). On the other hand the midline crossing of commissural axons also depends on the divergent receptor Robo3. By opposing the repulsion caused by Robo1 and Robo2, Robo3 promotes midline crossing (Sabatier et al., 2004; Friocourt and Chédotal, 2017). The ability of Robo3 to inhibit repulsion is not associated with competitive binding of Slit. This is because mammalian Robo3 has the ability to bind to Slit2 with significantly lower affinity compared to Robo1 (Zelina et al., 2014). Additionally, Robo3-dependent midline crossover loss is only partially recovered in Robo3/Slit1/Slit2 triple knockouts and Robo1/2/3 triple knockouts, suggesting that Robo3 likely functions in complementary pathways apart from Robo1 and Robo2, enabling midline crossing (Sabatier et al., 2004; Jaworski et al., 2010). Indeed, the Robo3 receptor does form a complex with netrin receptor DCC to enhance Netrin-DCC signaling. This requires tyrosine phosphorylation of Robo3 in its intracellular domain by Src-family kinases upon Netrin1 stimulation (Zelina et al., 2014). This finding shows Robo3’s functional importance as a DCC co-receptor, although it is still unclear how Netrin1 stimulation causes Robo3 to become phosphorylated and what signaling pathways are activated. Mutations in the Netrin receptor DCC have been identified in patients with CC agenesis. As mentioned above, this receptor is also involved in the second phase of CC development.

#### Similarity to other commissures

Multiple studies have reported that many vertebrate species express the same factors in the midline. For example, the localized expression of diffusible morphogens Wnt/BMP on the dorsal midline, Shh on the ventral midline, and Fgf on the neural crest are characteristics that humans share with many other vertebrates, including even lampreys. Members of the Wnt family are required for the formation of all forebrain commissures. They act via canonical Frizzled3-mediated transmission pathways and non-canonical, through tyrosine kinase receptors (Ryk) (Bagri et al., 2002; Gunhaga et al., 2003; Hébert et al., 2003; Andrews et al., 2006; Keeble et al., 2006; Fernandes et al., 2007; Lindwall et al., 2007; Geng et al., 2008; Jeong et al., 2008; Islam et al., 2009; Suárez et al., 2014a,b). The described morphogens form expression gradients that guide commissural axons in the right direction. An example of this is BMP7, whose expression initially prevents axonal growth into the interhemispheric line, however, later expression of Wnt3, regulated through GDF5 allows further growth and formation of midline structures (Shimogori et al., 2004; Choe et al., 2012). All forebrain commissures, including CC, initially cross the midline within a distinct anatomical region termed the commissural plate. In mice, four distinct molecular subdomains of the commissural plate have been identified, through which distinct commissural projections pass. Expression of the secreted protein *Fgf8* is crucial in the initial patterning of the forebrain and subsequent development of the commissural plate, and appears to act as an upstream regulator of many midline patterning molecules (Ohkubo et al., 2002; Storm et al., 2006; Hayhurst et al., 2008; Okada et al., 2008) that correlate anatomically with specific commissures (Moldrich et al., 2010). Dorsally, the CC passes through an *Emx1-and Nfia*-expressing domain; the hippocampal commissure passes through domains expressing *Nfia*, *Zic2* and *Six3*, and the anterior commissure passes through a *Six3*-expressing domain in the septum. Perturbed development of these subdomains results in disruption of the corresponding commissural projections passing through the domains, suggesting that correct patterning of the commissural plate is a prerequisite for commissure formation and midline cross (Moldrich et al., 2010).

#### Pioneer axons

The first, so-called «pioneer» axons of the cingulate gyrus also play an important role in the navigation of callosal axons during the development of the CC (Figure 5). They originate in the cingulate cortex and are the first to cross the midline, forming a pathway for subsequent commissural axons. It is believed that callosal axons interact directly with «pioneer» axons and follow the paved path. The axon-expressed ephrin receptor EphA3 has also been shown to mediate this axon-axon interaction (Koester and O'Leary, 1994; Deng and Elberger, 2001; Piper et al., 2009; Nishikimi et al., 2013).

### Fourth step: post-crossing and targeting

There is a mechanism that prevents callosal axons from re-entering the midline after crossing. Interaction between two pathways is important for this process. Interaction between Sema3C and its receptor Nrp1 initiates the process of attraction of pioneer axons toward the midline. However, after midline crossing, the Nrp1/Sema3C signaling is suppressed by EphrinB1 (Mire et al., 2018; Martín-Fernández et al., 2022). Pre-crossing CC axons express Nrp1 and are responsive to Sema3C, contrary to EphrinB1 whose expression is absent in these axons. Immediately after midline crossing, EphrinB1 expression increases, which prevents Sema3C to exert its attraction activity on post-crossing axons (Mire et al., 2018).

After callosal axons leave the midline, they target the same areas in the contralateral hemisphere they originate from. Molecular mechanisms that control correct area targeting are not very well understood. Experiments have shown that with a decrease in the excitability of neurons in layers II and III as a result of Kir2.1 overexpression, axon branching is reduced and patterns of CC innervation in the contralateral hemisphere are affected (Suárez et al., 2014b). However, this does not affect the identity of neurons, their migration or their ability to project axons (Mizuno et al., 2010; Hand et al., 2015). NMDAR has been shown to be important for both midline axonal crossing and area targeting. This receptor is expressed in the axons that cross the midline, and when inhibited or knocked down, a decrease in axonal crossing has been observed. Precise optogenetic stimulation of callosal neurons abolished the effects of NMDAR blockade, proving that NMDAR functioned via an activity-dependent mechanism. In turn, NMDAR impacts the expression of ARXA, a crucial gene for brain development linked to a number of neurological illnesses in humans. These findings highlight the important role played by NMDAR in the early decisions that control neural activity to direct axons (Gao et al., 2018). On the other hand, more recent experiments have also demonstrated the role of NMDAR in contralateral axon navigation. It turned out that inhibition of NMDAR, containing the GluN2B subunit, leads to a significant decrease in expression of EphB2, demonstrating a connection between NMDAR and the Ephrin-B/EphB system. Probably, Ephrin-B1, expressed in axons, transmits a signal to NMDAR through EphB2, located on target neurons, thereby regulating axons targeting in contralateral hemispheres (Zhou et al., 2021).

UL specific transcription factor Cux1 was also found to control area targeting by callosal axons. Cux1 down-regulation induces the impairment of the innervation of CC axons in the contralateral CP (Lefort et al., 2009; Rodríguez-Tornos et al., 2016). However, molecular mechanism downstream of Cux1 that controls interactions between the growth cone and target area are still to be uncovered.

## Conclusion remarks

Development of the largest commissure in the humans and apes, the corpus callosum, is a complex multistep process. Although, many molecular players have been identified in the last two decades, there is still a lot to be discovered. One of the most challenging questions: how do neocortical neurons that are born and reside in the same microenvironment of the SVZ/IZ, select one of the many alternative directions to send their axons? What is the role of SVZ/IZ environment in the process? How do CPN axons select to follow pioneer axons unidirectionally? One possibility is that there are gradients of either secreted or membrane bound molecular guidance cues in the SVZ/IZ that can be interpreted by callosal and non-callosal axons differently. There are several potential sources of such cues. It could be Tbr2 expressing intermediate progenitors or cells that just left mitotic cycle and started to differentiate. Pioneer callosal axons or incoming thalamocortical axons can also be a source of such gradients. It is also possible that corticofugal axons that appear before callosal axons can serve as a scaffold for them to navigate along their fibers in the opposite direction. Another potential source of guiding signals could be interneurons that migrate tangentially at the upper border of the SVZ from ganglionic eminences. Identification of the source (s) and molecular players that act as guiding cues for callosal axons would be a challenging task in the next decade.

Another thrilling issue, is how did the corpus callosum appear in evolution and what is its evolutionary advantage over the anterior commissure? The CC is present in placental mammals only, but absent in marsupials and monotremes, which use the evolutionary older anterior commissure to interconnect the two hemispheres of the forebrain. One could consider marsupials as alternative laboratory animals to investigate the evolutionary origin of corpus callosum with studies of comparative neuroanatomy. Animals such as opossums or fat-tailed dunnarts are good candidates for this role. The brain size and structure of the neocortex of both species are very similar to that of laboratory rodents. Both species are small, with opossum having the size of the rat, and fat-tailed dunnart being smaller than the mouse. They can be maintained in a standard animal facility and can be mated several times during a year. They can be used for comparative genomics and transcriptomic studies.

One possibility is that some of the genes that are crucial for callosal axons navigation and targeting, have changed their expression in placental animals during evolution. Therefore, it would be interesting to compare activity of enhancers of such genes between placental and non-placental mammals. On the other hand comparison of transcriptomes of neocortical cells with single cell resolution would help identifying them too. Once such genes or enhancers are identified, one could inactivate them or ectopically insert them in the mouse or laboratory marsupials. We think that these questions will soon be addressed with the help of new technologies.

## Author contributions

VT: Conceptualization, Supervision, Validation, Writing – review & editing. MG: Visualization, Writing – original draft, Writing – review & editing, Supervision. AK: Writing – original draft, Writing – review & editing, Visualization, Supervision. JC: Writing – original draft, Writing – review & editing, Supervision. PB: Visualization, Writing – original draft, Conceptualization. NM: Visualization, Writing – original draft.
